# Men having sex with men serosorting with casual partners: who, how much, and what risk factors in Switzerland, 2007-2009

**DOI:** 10.1186/1471-2458-13-839

**Published:** 2013-09-11

**Authors:** Stéphanie Lociciro, André Jeannin, Françoise Dubois-Arber

**Affiliations:** 1 Institute of Social and Preventive Medicine (IUMSP), Lausanne University Hospital, Biopôle 2, Route de la Corniche 10, 1010 Lausanne, Switzerland

**Keywords:** Sexual risk behaviour, Men who have sex with men, Serosorting, HIV, Switzerland

## Abstract

**Background:**

Serosorting is practiced by men who have sex with men (MSM) to reduce human immunodeficiency virus (HIV) transmission. This study evaluates the prevalence of serosorting with casual partners, and analyses the characteristics and estimated numbers of serosorters in Switzerland 2007-2009.

**Methods:**

Data were extracted from cross-sectional surveys conducted in 2007 and 2009 among self-selected MSM recruited online, through gay newspapers, and through gay organizations. Nested models were fitted to ascertain the appropriateness of pooling the datasets. Multiple logistic regression analysis was performed on pooled data to determine the association between serosorting and demographic, lifestyle-related, and health-related factors. Extrapolations were performed by applying proportions of various types of serosorters to Swiss population data collected in 2007.

**Results:**

A significant and stable number of MSM (approximately 39% in 2007 and 2009) intentionally engage in serosorting with casual partners in Switzerland. Variables significantly associated with serosorting were: gay organization membership (aOR = 1.67), frequent internet use for sexual encounters (aOR = 1.71), having had a sexually transmitted infection (STI) at any time in the past 12 months (aOR = 1.70), HIV-positive status (aOR = 0.52), regularly frequenting sex-on-premises venues (aOR = 0.42), and unprotected anal intercourse (UAI) with partners of different or unknown HIV status in the past 12 months (aOR = 0.22). Approximately one-fifth of serosorters declared HIV negativity without being tested in the past 12 months; 15.8% reported not knowing their own HIV status.

**Conclusion:**

The particular risk profile of serosorters having UAI with casual partners (multiple partners, STI history, and inadequate testing frequency) requires specific preventive interventions tailored to HIV status.

## Background

A renewal of the human immunodeficiency virus (HIV) epidemic has been observed among men who have sex with men (MSM) in developed countries [1]. In Switzerland in 2010, although the proportion of recent infections (i.e., according to laboratory test methods, diagnosed within 6 months after the infection has occurred) had been decreasing among newly declared infections in MSM since 2008, the proportion of overall new HIV-positive tests among MSM was still increasing to reach about 47% of all declared infections [2].

The increase in HIV testing may be one explanation for the re-emergence of the HIV epidemic among MSM [3]. However, in Switzerland, between 1994 and 2009, we observed a stable proportion of respondents reporting having been tested during the last 12 months, and an increase of 10 points in respondents having had at least one incident of unprotected anal intercourse (UAI) during the past 12 months with a partner of different or unknown HIV status [4].

Risk reduction practices other than condom use have been extensively studied [5-9]. Serosorting - choosing to have UAI with partners of the same HIV status–has been specifically studied [10-15], and has been considered to have a protective effect or convey a lower risk of HIV transmission in populations with a high prevalence and frequency of HIV testing [16-19]. However, the limits of this approach have also been demonstrated: serosorting may increase HIV transmission in populations with high rates of unrecognized and/or acute infection [10,20].

Serosorting can be perceived as a marker of freedom for MSM living with HIV, allowing them to believe that they can have unprotected sex without considering HIV transmission. However, it does not prevent the transmission of other sexually transmitted infections (STIs) [5], and disclosing one’s own HIV positivity may be difficult.

For HIV-negative MSM, serosorting still carries the risk of being infected with HIV. First, serostatus may not be truthfully declared, either with a steady partner or with a casual partner. Authors highlighted that a majority of new HIV infections occurred within steady relationships [21]. Next, the knowledge of one’s own or one’s partner’s HIV status may be inaccurate. Williamson et al. estimated that 41% of HIV-positive MSM enrolled in their study believed themselves to be HIV-negative [22]. One may genuinely believe himself to be HIV-negative, having had their last HIV test during the primary phase of infection within the seroconversion window [23].

This observational study focuses on the intentional practice of serosorters among MSM living in Switzerland who had anal intercourse with casual partners during the past 12 months without using a condom. The aims were:

a) to evaluate the prevalence of the practice in 2007 and 2009;

b) to analyse the characteristics of these specific serosorters; and

c) to estimate by extrapolation the number of MSM at risk of contracting HIV or other STIs in Switzerland as a result of serosorting with casual partners.

## Methods

### Study population and data collection

Data were obtained from the 2007 (N = 2953) and 2009 (N = 1929) Swiss Gaysurvey, a repeated (nine times between 1987 and 2009) cross-sectional survey conducted in self-selected samples of MSM living in Switzerland. Respondents are recruited online with banners published on the main gay websites within the Switzerland Internet domain (“ch”), and through gay newspapers and gay or HIV/AIDS nongovernmental organizations (paper-and-pencil version of the questionnaire).

The survey (pertaining to the Swiss HIV/STI behavioural surveillance system) used an anonymous self-administered questionnaire. The questionnaire has been used in its current form for most items since 1992. The main indicators that are used for surveillance were agreed upon at European level [24]. The items on sexual risk reduction practices were first introduced in the core questionnaire in 2007. The data collection methodology and the practical details of the questionnaire have been already presented elsewhere [25].

The questionnaire was reviewed by the Swiss Federal Office of Public Health, the Swiss Aids Foundation, and gay community leaders. The survey was approved by the ethical review board of the Faculty of Medicine and Biology at Lausanne University, Switzerland.

The questionnaire provided information about socio-demographic characteristics, sexual activity, HIV status (self-report), STI history, and preventive behaviours in different contexts of relationships (casual/steady partners and partners of different or unknown HIV status). The practice of serosorting was assessed with one question, referring to UAI with casual partners and stressing the participant’s intention to reduce HIV transmission risks:

“Over the past 12 months, did you ever practice anal intercourse without a condom and ask your partner if he was of the same HIV-status as you, in order to avoid HIV infection:”

– *with steady partner (yes/no).*

– *with casual partners (yes/no).*

This question did not differentiate between insertive and receptive anal intercourse, and a casual partner was defined in the questionnaire as any sexual partner that the participant did not consider to be his steady partner. The word “serosorting” was not used in the questionnaire.

### Population

Inclusion criteria for the studied population were: having had sex with a man at least once, having had a casual partner in the past 12 months, and having had anal intercourse without a condom with at least one casual partner in the past 12 months.

### Statistical analysis

We proceeded first to a statistical description of the 2007 and 2009 data sets. Trends between these two years were tested with the chi-squared test of goodness of fit. To minimize Type I error, the significance level was determined using the Bonferroni adjustment procedure (α-level=0.003), yielding an overall level of 0.5.

Multiple logistic regression analysis was performed to study the interaction effects of interview mode and year of the survey with variables of interest. Interaction terms were built in order to make a decision concerning the possibility of merging the paper and web data sets, as well as the 2007 and 2009 data sets.

The dependent variable was “to have practiced serosorting over the past 12 months” and the following variables were used as regressors: survey years (2007 vs. 2009), survey mode (paper vs. online questionnaire), age (< 25; 25-49; ≥ 50 years of age), university degree, nationality, residence area (more than 100,000 inhabitants), membership in a gay organization, to currently have or having had a steady partner during the past 12 months, number of sexual partners with anal intercourse (AI) during the past 12 months (dichotomized at median of 6), regular visiting of sex-on-premises venues, frequent use of the internet for sexual encounters during the past 12 months, any STIs during the past 12 months, UAI with a partner of different or unknown HIV status during the past 12 months, an HIV test during the past 12 months, HIV status declared, and frequent substance use while having sex during the past 12 months.

To ascertain whether it was appropriate to pool the two datasets, three nested models were fitted: Model 1 included all factors plus the two selected interaction terms; Model 2 included all factors plus interaction terms with ‘survey mode’; Model 3 included all factors plus interaction terms with ‘survey years’. Likelihood-ratio tests were performed after the logistic regressions to compare Models 2 and 3 with Model 1.

Next, multiple logistic regression analysis was performed on pooled data to determine the association between serosorting and demographic, lifestyle-related, and health-related factors. Odds ratios and 95% confidence intervals were calculated for each of predictor mentioned above.

Finally, the number of MSM at various levels of risk for HIV as a result of serosorting with casual partners was estimated through extrapolation by applying proportions of different types of serosorters found in the pooled Gaysurveys 2007 and 2009 to Swiss population data collected in the Swiss Health Survey 2007. This survey defines MSM on the basis of self-reported types of sexual partners interacted with during one’s lifetime, using a modified Kinsey indicator [26]. Two definitions of MSM were chosen: a restrictive definition that includes men who have sex only with men, mainly with men, and with as many men as women (N_Swiss_ = 33,700); and a more inclusive definition that includes all men who have had sex with a man at least once in their lifetime (N_Swiss_ = 70,300). We estimated several proportions of MSM at risk in Switzerland–with their 95% confidence interval–according to different types of situations. To define these diverse risk situations, we used the following variables: had an HIV test during the past 12 months, and (for HIV-positive MSM) the viral load and occurrence of STIs in the past 12 months. Confidence intervals were obtained for each proportion and used to evaluate the minimum and maximum number of MSM involved for each type of situation.

Data were analysed using the statistical package STATA 11.1 (StataCorp LP, College Station, Texas, USA).

## Results

### Population

**Figure 1:**
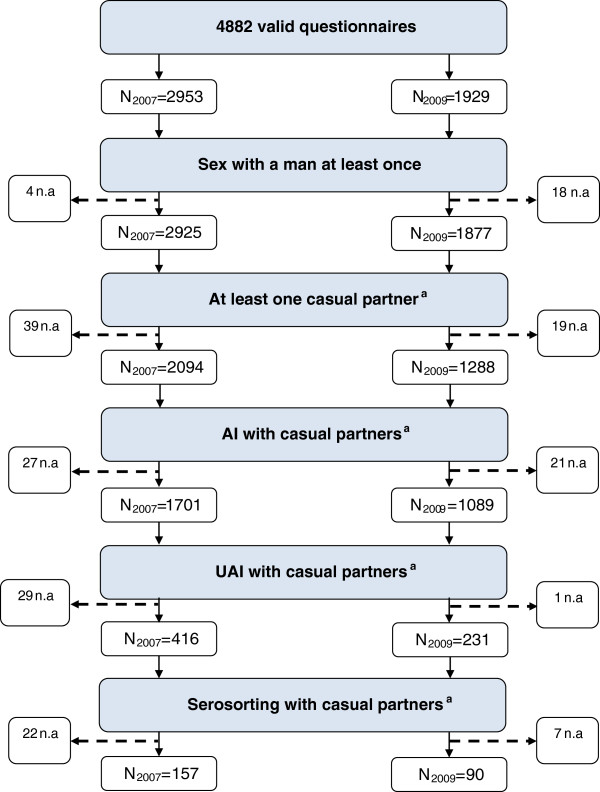
Filters applied for analyses: population under study.

Figure 1 presents the details for each survey year with different filters applied. Of 4882 MSM who answered in 2007 and 2009 (aggregated), 13.3% (n = 647) have had UAI with casual partners in the past 12 months. Among them, 38.2% (N = 247) used serosorting as a harm reduction practice.

### Respondents’ characteristics

Respondents who had UAI with casual partners during the past 12 months (N_2007_ = 416; N_2009_ = 231) are presented in Table 1. Approximately two-fifths practiced serosorting (2007: 37.7%; 2009: 39.0%). This difference was not statistically significant. The two samples varied little: there were more non-Swiss nationals in 2009 (27.7%) than in 2007 (16.6%, p = 0.001), and the proportion of respondents reporting an STI in the past 12 months was higher in 2009 (2009: 24.2%; 2007: 17.3%, p = 0.003).

**Table 1:** **Univariate analysis: characteristics of participants**^†^

	**2007**		**2009**		**P-value**	**Pooled data**	
	**N = 416 (%)**		**N = 231 (%)**			**N = 647 (%)**	
**Serosorting**					0.412		
Yes	157	(37.7)	90	(39.0)		247	(38.2)
No	237	(57.0)	134	(58.0)		371	(57.3)
No answer	22	(5.3)	7	(3.0)		29	(4.5)
**Age**					0.200		
< 25 yr.	82	(19.7)	49	(21.2)		131	(20.2)
25–49 yr.	282	(67.8)	150	(64.9)		432	(66.8)
≥ 50 yr.	46	(11.1)	32	(13.9)		78	(12.1)
No answer	6	(1.4)	0	-		6	(0.9)
**Survey mode**					0.100		
Paper	149	(35.8)	68	(29.4)		217	(33.5)
Internet	267	(64.2)	163	(70.6)		430	(66.5)
**University degree**					0.574		
Yes	185	(44.5)	110	(47.6)		295	(45.6)
No	230	(55.3)	121	(52.4)		351	(54.3)
No answer	1	(0.3)	0	-		1	(0.2)
**Nationality**					**0.001**		
Swiss national	336	(80.8)	166	(71.9)		502	(77.6)
Non Swiss national	69	(16.6)	64	(27.7)		133	(20.6)
No answer	11	(2.6)	1	(0.4)		12	(1.9)
**Residence area > 100,000 inhabitants**					0.297		
Yes	174	(41.8)	86	(37.2)		260	(40.2)
No	241	(57.9)	143	(61.9)		384	(59.4)
No answer	1	(0.2)	2	(0.9)		3	(0.5)
**Gay organization**					0.602		
Yes	60	(14.4)	38	(16.5)		98	(15.1)
No	355	(85.3)	193	(83.6)		548	(84.7)
No answer	1	(0.2)	0	-		1	(0.2)
**Steady partner**^**a**^					0.038		
Yes	172	(41.4)	116	(50.2)		288	(44.5)
No	240	(57.7)	115	(49.8)		355	(54.9)
No answer	4	(1.0)	0	-		4	(0.6)
**Number of sexual partners with AI**^**a**^					0.219		
None	9	(2.2)	2	(0.9)		11	(1.7)
1–5 partners	200	(48.1)	102	(44.2)		302	(46.7)
≥ 6 partners	207	(49.8)	126	(54.6)		333	(51.5)
No answer	0	-	1	(0.4)		1	(0.2)
**Regularly frequenting sex-on-premises venues**^**a**^					0.659		
Yes	82	(19.7)	47	(20.4)		129	(19.9)
No	322	(77.4)	180	(77.9)		502	(77.6)
No answer	12	(2.9)	4	(1.7)		16	(2.5)
**Frequent use of the internet for sexual encounters**^**a**^					0.558		
Yes	178	(42.8)	109	(47.2)		287	(44.4)
No	232	(55.8)	119	(51.5)		351	(54.3)
No answer	6	(1.4)	3	(1.3)		9	(1.4)
**Sexually transmitted infections**^**a**^					**0.003**		
Yes	72	(17.3)	56	(24.2)		128	(19.8)
No	330	(79.3)	175	(75.8)		505	(78.1)
No answer	14	(3.4)	0	-		14	(2.2)
**UAI with partners of different or unknown HIV status**^**a**^					0.510		
Yes	295	(70.9)	155	(67.1)		450	(69.6)
No	118	(28.4)	75	(32.5)		193	(29.8)
No answer	3	(0.7)	1	(0.4)		4	(0.6)
**HIV test**^**a**^					0.087		
Yes	177	(42.6)	106	(45.9)		283	(43.7)
No	231	(55.5)	125	(54.1)		356	(55.0)
No answer	8	(1.9)	0	-		8	(1.2)
**HIV status declared**					0.021		
Unknown	94	(22.6)	62	(26.8)		156	(24.1)
HIV negative	256	(61.5)	117	(50.7)		373	(57.7)
HIV positive/Aids	66	(15.9)	52	(22.5)		118	(18.2)
**Frequent substance use while having sex**^**a**^					0.054		
Yes	119	(28.6)	83	(35.9)		202	(31.2)
No	297	(71.4)	148	(64.1)		445	(68.8)

^**†**^ Based on inclusion criteria: UAI with casual partners in the past 12 months.

^a^ in the past 12 months.

^b^ Among respondents who had a steady partner and had AI with him in the past 12 months (N_2007_ = 138 and N_2009_ = 116.)

*AI* Anal Intercourse, *UAI* Unprotected Anal Intercourse.

### Multivariate analysis: predictors of serosorting

#### Non-responses management

Non-responses rates were inferior or equal to 5% for all variables (Table 1), and were therefore merged with respondents having answered ‘No’ to the question for the multivariate analysis. Regarding the ‘age’ and ‘number of sexual partners with UAI’ variables, non-responses were replaced with median age (36 years) and median number of partners (2 partners).

#### Logistic regression models and LR tests

Multivariate logistic regression was run for Models 1, 2, and 3. No significant interactions were observed according to the LR test between Models 1 and 2 (LR χ^2^ (14) = 14.6; p = 0.406) or between Models 1 and 3 (LR χ^2 ^(14) = 18.1; p = 0.202). Adding interaction terms as predictor variables did not result in a statistically significant improvement in model fit. Thus, adding mode and year interaction terms as predictor variables did not result in a statistically significant improvement in model fit and a final, simpler model (Model 4) composed exclusively of regressors without any interaction terms was retained. On this basis, the two samples (2007 and 2009) were pooled for further analysis.

Six variables were significantly associated (p < 0.05) with serosorting in this final Model 4 (Table 2). Gay organization membership (aOR = 1.67), frequent internet use for sexual encounters (aOR = 1.71), and having had an STI in the past twelve months (aOR = 1.70) were factors positively associated with serosorting. Regarding reported HIV status, only positive HIV status was significantly and negatively associated with serosorting (aOR = 0.52). Two other factors were significantly negatively associated with serosorting: regularly frequenting sex-on-premises venues (aOR = 0.42), and UAI with partners of different or unknown HIV status in the past 12 months (aOR = 0.22).

**Table 2:** Multivariate logistic regression: factors associated with serosorting

	**aOR**	**95% CI**	**P-value**
**Survey year**			
2007	1		0.982
2009	1.00	0.69–1.46	
**Survey mode**			
Paper	1		
Internet	0.83	0.55–1.25	0.367
**Age**			
< 25 yr.	0.83	0.50–1.36	0.457
25–49 yr.	1		
≥ 50 yr.	0.72	0.40–1.30	0.275
**University degree**			
Yes	0.90	0.61–1.32	0.579
No	1		
**Nationality**			
Non-Swiss national	0.98	0.62–1.55	0.943
Swiss national	1		
**Residence area > 100,000 inhabitants**			
Yes	1.22	0.84–1.77	0.306
No	1		
**Gay organization**			
Yes	**1.67**	**1.02–2.73**	**0.043**
No	1		
**Steady partner**^**a**^			
Yes	0.75	0.52–1.08	0.119
No	1		
**Number of sexual partners with AI**^**a**^			
1–5 partners	1		
≥ 6 partners	1.32	0.88–1.99	0.181
**Regularly frequenting sex-on-premises venues**^**a**^			
Yes	**0.42**	**0.26–0.708**	**0.001**
No	1		
**Frequent use of the internet for sexual encounters**^**a**^			
Yes	**1.71**	**1.16–2.50**	**0.006**
No	1		
**Sexually transmitted infections**^**a**^			
Yes	**1.70**	**1.07–2.69**	**0.024**
No	1		
**UAI with partners of different or unknown HIV status**^**a**^			
Yes	**0.22**	**0.14–0.32**	**0.000**
No	1		
**HIV test **^**a**^			
Yes	1.47	0.98–2.19	0.063
No	1		
**HIV status declared**			
Unknown	0.74	0.44–1.24	0.256
HIV negative	1		
HIV positive/Aids	**0.52**	**0.31–0.87**	**0.013**
**Frequent substance use while having sex**^**a**^			
Yes	1.01	0.67–1.52	0.958
No	1		

The reference category for the dependant variable is “having practised serosorting”. Bold denotes adjusted odds ratio significant at the 0.003 level.

^a^ In the past 12 months.

^b^ Among respondents who had a steady partner and had AI with him in the past 12 months (N_2007_ = 151 and N_2009_ = 117).

AI: Anal Intercourse; aOR: adjusted odds ratio; CI: confidence interval.

### Estimates of the number of MSM at risk of contracting HIV in Switzerland as a result of serosorting with casual partners

Because the year and survey mode did not provide additional information about the practice of serosorting, data from 2007 and 2009 and the paper and web surveys were pooled for this analysis (N = 4882) to further investigate the practice with respect to the respondent’s HIV status, and (for HIV-positive respondents) current viral load and STI occurrence, during the past 12 months. Among the 1929 respondents in 2009, 517 (27%) had participated in the 2007 survey.

Table 3 presents the extrapolated numbers of MSM at risk of contracting HIV as a result of serosorting in Switzerland. They have been determined according to the percentage and confidence interval of MSM concerning different scenarios within the pooled Gaysurvey data. The mean number of sexual partners with whom respondents have had AI during the past 12 months was calculated and presented with standard deviation to develop a picture of the number of respondents potentially concerned by this risk-taking. Respondents who reported having more than 80 partners were considered outliers and excluded from this calculation.

**Table 3:** Serosorting: extrapolated country MSM population

	**Gaysurvey sample data**							**Extrapolated to country MSM population (17–74 yr.)**			
								**a) Restricted definition (sex only with men, mainly with men, and with as many women as men, lifetime)**		**b) Enlarged definition (at least one partner of same sex, lifetime)**	
	**N**	** %**	**CI 95% **^**c**^			**% Among Serosorters %**	**Mean number of sexual partners with AI**^**d**^	**N**	**Min**_**a**_**/max**_**a**_	**N**	**Min**_**b**_**/max**_**b**_
Pooled Gaysurvey data (2007-2009)	4882	100						33,700			70,300
UAI with casual partners^a^	647	13.3	12.3	-	14.2		11 ± 1	4466	4150/4798	9317	8656/10,008
Serosorting with casual partners^b^	247	5.1	4.5	-	5.7	N = 247	11 ± 1	1705	1503/1925	3557	3136/4015
Serostatus declared											
HIV-positive	44	0.9	0.7	-	1.2	17.8	18 ± 2	304	221/407	634	461/849
Detectable viral load and No STI^e^	10	0.2	0.1	-	0.4	4.0	17 ± 5	69	33/127	144	69/265
Undetectable viral load and No STI	15	0.3	0.2	-	0.5	6.1	18 ± 4	104	67/169	216	141/352
Undetectable viral load and STI	12	0.2	0.1	-	0.4	4.9	15 ± 4	83	43/145	173	89/302
Detectable viral load and STI	7	0.1	0.1	-	0.3	2.8	24 ± 5	48	19/99	101	41/208
HIV-negative	164	3.4	2.9	-	3.9	66.4	10 ± 1	1132	968/1316	2362	2019/2744
Tested in the past 12 months	112	2.3	1.9	-	2.8	45.3	11 ± 1	773	638/928	1613	1331/1936
Not tested in the past 12 months	52	1.1	0.8	-	1.4	21.1	8 ± 1	359	268/470	749	560/980
N.A, D.K, Not tested^f^	39	0.8	0.6	-	1.1	15.8	10 ± 2	269	192/367	562	400/767

a: Unprotected anal intercourse (UAI) in the past 12 month.

b: Among respondents who had UAI with casual partners in the past 12 months (N = 647).

c: Confidence interval (CI) calculated with the exact binomial distribution.

d: Anal intercourse in the past 12 months.

e: Sexually transmitted infections (STIs) in the past 12 months.

f: N.A: No answer; D.K: Don’t know.

The extrapolated data show that a minimum of 4150 and a maximum of 10,008 MSM have had UAI with a casual partner in the past 12 months, among whom between 1503 and 4015 practiced serosorting.

Of all serosorters, 17.8% (221 < N < 849) reported being HIV-positive. They were analysed according to two parameters that would represent increased risk of HIV transmission: reporting having had a detectable viral load (or not), and reporting having had an STI (or not). Between 19 and 208 MSM in Switzerland reported having had a detectable viral load and STI. Most of the HIV-positive serosorters had an undetectable viral load and no STI (67 < N < 352), i.e. no increased risk of HIV/STI transmission [27].

Of all serosorters, 66.4% (968 < N < 2744) declared that they were HIV-negative. They were considered from the perspective of being tested for HIV in the past 12 months (i.e., having more accurate knowledge of their own HIV status). Data indicated that 45.3% (638 < N < 1936) of the serosorters had been tested recently and were HIV-negative. However, 21.1% (268 < N < 980) declared that they were HIV-negative even though they had not been tested in the past 12 months, and another 15.8% (192 < N < 767) MSM serosorted while they had not been recently tested for HIV or did not know their HIV status (i.e., with a higher risk of potentially transmitting HIV to their casual partners).

The mean number of sexual partners was 18(± 2) among HIV-positive serosorters and 10(± 1) among HIV-negative serosorters; the data suggests that a large population of MSM are involved in this practice.

## Discussion

A significant and stable number of MSM (approximately 39% in 2007 and 2009) were classified as engaging in serosorting with casual partners in Switzerland. Estimates concerning the number of persons involved in various levels of risk were provided.

Risk reduction practices are often analysed in publications as several overlapping questions regarding respondents’ sexual behaviour: the question of UAI with steady and/or casual partners, paired with the presumed or proven serostatus of the respondent and the supposed serostatus of the respondent’s partners. Serosorting assumes that the protagonists have disclosed their respective HIV statuses beforehand with the explicit aim of avoiding HIV infection. However, this intention concept is often not made explicit in the serosorting definition or is entirely missing from questionnaires. The Swiss GaySurvey focused on risk reduction practices with steady and casual partners in the last two survey waves [4], and asked specifically whether the respondent acted with the purpose of preventing HIV transmission. The word “serosorting” itself was deliberately not used in the questionnaire in order not to influence the respondents.

Multivariate analysis tends to indicate that serosorting may be practiced as a structured, planned strategy, when we consider factors negatively and positively associated with serosorting. MSM who reported themselves as HIV-positive and that they have had UAI with partners of different or unknown HIV status are indeed less likely to engage in serosorting. Regularly visiting sex-on-premises venues is also negatively associated with serosorting. This negative association might be explained by the difficulty of disclosing one’s HIV status in places (e.g., backrooms, darkrooms, or saunas) where verbal interactions are not encouraged [28].

Serosorting was positively associated with belonging to a gay organisation, possibly owing to existing debates on risk reduction within these organisations in Switzerland, and more informed choices resulting from these discussions. Similarly, frequent use of the internet to select partners is associated with serosorting. Partner selection through the internet may seem an appropriate method a priori because it can be easier to declare one’s HIV status anonymously, rather than face-to-face, or it may simply be faster to find a partner of same HIV status. However, these conclusions contrast with findings from Berry et al., who showed that internet usage was significantly associated with an increased likelihood of UAI with potentially discordant partners among HIV-negative MSM [29].

As expected, our data revealed a positive association between serosorting and reporting an STI in the last 12 months [30]. Serosorting was much more practiced by HIV-negative men tested in the past 12 months than by HIV-positive MSM (45.3% vs. 17.8%). This result was expected because of the wording of the original question. However, 21.1% of serosorters declared themselves to be HIV-negative without having been tested during the past 12 months (268 < N < 980), and 15.8% reported not knowing their HIV status (192 < N < 767) and may be considered at risk of being infected with HIV or of infecting other people with HIV. This finding is disturbing, particularly regarding the high mean number of sexual partners reported.

The particular risk profile of these serosorters who have UAI with casual partners (multiple partners, UAI with partners of different or unknown status, STI history, and partially inadequate testing frequency) requires preventive interventions tailored to HIV status.

Our study focused on MSM who have casual male partners, and does not go into detail about any relationship with a steady partner among these men. Moreover, a certain proportion of MSM serosorters also have sex with women. We can make the assumption that the practice of serosorting carries a risk for both sexes, as well as for both homosexual and heterosexual couples. This component should also be taken into account within prevention programmes.

Our results confirm those of several authors, notably Heymer et al., who concluded that *serosorting has a real potential to increase risk and should not be promoted as a public-health strategy*[31]. HIV testing alone is not a panacea, and frequent testing for HIV and other STIs, behavioural interventions, and emphasis on primary infections should be jointly promoted.

Our study has limitations: Gaysurvey data are not representative of the entire MSM population. The broad dissemination of our questionnaire likely attenuated selection bias. However, this method may overestimate levels of risky behavior, given that several of the sites or newspapers used for recruitment are also used to contact partners. We also do not know how often serosorting occurs, or the absolute number of partners with whom serosorters engaged in serosorting. MSM who responded to Gaysurvey may be numerous to be concerned by serosorting; the intensity of risk remains unknown.

We did not exclude from the 2009 dataset those who reported having participated in 2007. The proportion of serosorters in 2009 who had reported having participated in the 2007 survey was not significantly different from the non-serosorters in this situation (respectively 23.2% and 25%). Furthermore, most of the variables associated with serosorting were variables measuring behaviours reported over a period of 12 months (last 12 months).

The quality of the extrapolation of the numbers of serosorters to the MSM population in Switzerland is dependent upon the quality of the GaySurvey samples used in the computations, which remains unknown. However, we also relied on data from a health survey in the general population, with a restricted and an enlarged definition of MSM, to compute these extrapolated estimates. That survey is a random probability survey which does not suffer from the same weaknesses as GaySurvey and is uncorrelated to it.

## Conclusion

Despite these limitations we feel that our study brings careful estimates that may be useful to plan preventive activities.

## Abbreviations

MSM: Men who have sex with men; HIV: Human immunodeficiency virus; STI: Sexually transmitted infection; UAI: Unprotected anal intercourse.

## Competing interests

The authors declare that they have no competing interests.

## Authors’ contributions

It is an original article–not submitted elsewhere–in which all the authors have contributed significantly: SL data collection, data analysis, writing of the article, AJ, survey conceptualization, data analysis supervision, participation in the writing and final approval, FDA, conceptualization and direction of the study, participation in the writing and final approval. All authors read and approved the final manuscript.

## Pre-publication history

The pre-publication history for this paper can be accessed here:

http://www.biomedcentral.com/1471-2458/13/839/prepub
